# Emergent dynamics of neuromorphic nanowire networks

**DOI:** 10.1038/s41598-019-51330-6

**Published:** 2019-10-17

**Authors:** Adrian Diaz-Alvarez, Rintaro Higuchi, Paula Sanz-Leon, Ido Marcus, Yoshitaka Shingaya, Adam Z. Stieg, James K. Gimzewski, Zdenka Kuncic, Tomonobu Nakayama

**Affiliations:** 1 International Center for Material Nanoarchitectonics (WPI-MANA), National Institute for Materials Science (NIMS), 1-1 Namiki, Tsukuba, Ibaraki 305-0044 Japan; 2 Sydney Nano Institute and School of Physics, University of Sydney, Sydney, NSW 2006 Australia; 3 California NanoSystems Institute (CNSI), University of California Los Angeles, 570 Westwood Plaza, Los Angeles, California 90095 USA; 4 Department of Chemistry and Biochemistry, University of California Los Angeles, 607 Charles E. Young Drive East, Los Angeles, California 90095 USA; 5 Graduate School of Pure and Applied Sciences, University of Tsukuba, 1-1 Namiki, Tsukuba, Ibaraki 305-0055 Japan

**Keywords:** Nanowires, Electronic devices, Materials science, Nanoscience and technology

## Abstract

Neuromorphic networks are formed by random self-assembly of silver nanowires. Silver nanowires are coated with a polymer layer after synthesis in which junctions between two nanowires act as resistive switches, often compared with neurosynapses. We analyze the role of single junction switching in the dynamical properties of the neuromorphic network. Network transitions to a high-conductance state under the application of a voltage bias higher than a threshold value. The stability and permanence of this state is studied by shifting the voltage bias in order to activate or deactivate the network. A model of the electrical network with atomic switches reproduces the relation between individual nanowire junctions switching events with current pathway formation or destruction. This relation is further manifested in changes in 1/f power-law scaling of the spectral distribution of current. The current fluctuations involved in this scaling shift are considered to arise from an essential equilibrium between formation, stochastic-mediated breakdown of individual nanowire-nanowire junctions and the onset of different current pathways that optimize power dissipation. This emergent dynamics shown by polymer-coated Ag nanowire networks places this system in the class of optimal transport networks, from which new fundamental parallels with neural dynamics and natural computing problem-solving can be drawn.

## Introduction

The human brain is a product of evolution, tuned and reshaped by an ever-changing environment. The brain’s neuronal system is able to achieve the ability to recognize, conceptualize and memorize objects in the physical world. Using environmental information we establish logical associations that ultimately allows us not only to survive, but also to solve highly complex problems^1^.However, in an increasingly connected and interactive world, the volume of information to process has exponentially increased, and in order to extract and synthesize meaningful information, computerized approaches, such as machine learning and its various incarnations have gained tremendous popularity^2^.

Typically, Artificial Neural Networks (ANNs) attain this goal by a very delicate and case-selective combination of learning strategies^3^. Data containing complex or contextual associations between objects normally requires an heuristic sampling which limits their ability to synthesize information. Conventional CMOS architectures also restrains the amount of data that is efficiently processed with ANNs due to power consumption bottlenecks.

Interest in the creation of synthetic neurons that could increase the processing abilities of ANNs has increased considerably with the discovery of nanomaterials with memristive properties^4^. A memristive device is a non-linear two-terminal device in which the resistance shows resilience to change (i.e. memory), manifested in hysteretic behavior when the energy change is reversed or reduced, also termed as resistive switching. The memristor thus has two important neurosynapse-like properties, plasticity and retention. Traditional integrate-and-fire models, that emulate the electrical behavior of neurons using passive circuit elements, can be simulated exclusively with these elements^5–7^. Memristive devices have been successfully embedded into various CMOS architectures, enabling the realization of synthetic neural networks(SNN). SNNs imitate the topology of an ANN in a physical layout, typically stacking memristive terminals in cross-bar configurations^8,9^. Using voltage pulses to configure the internal state, or *weight*, of individual memristors; memorization, learning and classification abilities have been achieved^10–13^. However promising, this approach remains reliant upon CMOS technology and inherits some of its limitations: large cost-efficiency ratio, high power consumption, and subpar performance with respect to computerized ANNs.

Neuromorphic networks offer a different approach to synthetic neural networks; rather than focusing on the controllability of single synthetic neurons to replicate the learning schemes of ANN, they focus on mimicking the complex network topology of neurons in the brain by creating similarly complex networks comprising nanomaterials such as nanowires or nanoparticles. The contact areas between resistive elements in a neuromorphic network show resistive switching properties similar to those of memristors or atomic switches^14–17^.

Atomic Switch Networks (ASN) made of Ag_2_S junctions present these properties^18,19^. ASNs are created through a combination of bottom-up synthesis and top-down lithographic patterning. A grid of copper posts is used as a seed for decomposition of silver form solution, resulting in the growth of dendritic networks of Ag nanowires whose interconnections, following sulfurization, act as atomic switches. This bottom-up fabrication replicates the high-density and intertwined connectivity of real biological neurons. The exponential increase in neurosynapse-like junctions adds a layer of topological complexity to the network, enhancing its plasticity and adaptability. Demonstrations of natural computing paradigms such as reservoir computing have been reported, using the networks to perform Boolean operations or signal reconstructions^20–23^.

Alternative approaches to constructing neuromorphic networks relies on bottom-up formation to create random self-assembled networks of nanowire or nanoparticles. Brown and coworkers have created nanoparticle networks by sputtering cluster of different metal oxide nanoparticles, like gold^24^ or tin^25^, in a controlled atmosphere. The individual nanoparticle junctions show properties of metal oxide resistive switches, with tunneling and filament formation in the switches, conferring neuromorphic properties to the networks^26^. They also found some evidence of recurrent properties such as critical activation, memorization and stochastic-mediated dynamics^24,27,28^.

Many metallic nanowire or nanoparticle compounds can be synthesized in solution, so the formation of a disperse network is achieved with simpler solution-process techniques, such as drop casting or spray coating^29^. Factors such as density and homogeneity can be tuned by changing solution concentration or droplet size. Ag nanowires are amongst the most widely used to build metallic networks due to their low resistance^30^, low cost and elasticity, which have found several applications as flexible transparent electrodes^29,31^. Synthesis of silver nanowires follows a particular reaction process called the polyol process, in which a polyol based solution precludes the oxidation of silver nitrates. Adding polymer species to the solution such as polyvinilpirrolydone (PVP), which selectively attaches to particular facets of silver aggregates, promotes the formation of Ag nanoparticles or nanowires with polymer coated surfaces^32–35^. PVP coating is insulating, so disposal of this layer is fundamental to form low resistance networks^30^. Surprisingly, however, PVP-Ag nanowire-nanowire or nanoparticle-nanoparticle junctions show resistive switching properties^36,37^. This can be attributed to the growth of metal filaments between the nanowires, possibly facilitated by the ionic transport of silver through the PVP layer after the application of a high-electric field across the polymeric junction between two nanowires. Resistive switching behavior originated by metallic filament growth has also been reported for polymer-based electrolytic atomic switches^38^ and in composites of Ag nanowire networks embedded in polymer matrices^39^. In a nanowire network fed by an external current or voltage source, resistive switching in individual junctions produce a dynamic tuning of the effective resistance of the whole network. Formation of low resistance pathways between the probes contacting the networks induces a transition from a low-conductance state(LCS) to a high-conductance state(HCS) at a given voltage threshold. Altering network density and controlling probe spacing can affect this threshold(or activation threshold), but it is routinely found at less than 10 V, even for millimetric space between probes and densities down to 0.08 nanowires/μm^2^ ^40^.

In the present work, randomly self-assembled PVP-Ag nanowire networks are created in solution and drop-casted onto a SiO_2_ substrate. Activation threshold of these networks and the role of resistive switching of single nanowire-nanowire junctions in the electrical properties has been studied previously^30,36,40,41^. However, spatio-temporal correlations are ubiquitous in ASNs^18,42^. This correlations are translated to power-laws in the power spectral distribution (PSD) of signals going through the network. Many dynamical processes showing these power-laws are always present in system in the brink of criticality^43^, such as those found in brain waves^44^. We have inspected this correlational dynamics in PVP-Ag networks. Current-time series are acquired on networks connected by two fixed probes controlled by a bias voltage. Current fluctuation and evolution during the time series is compared with an electrical model of the network. In the model, nanowire-nanowire junctions are considered as filament-like atomic switches. Network activation is inspected under a constant bias voltage high enough to guarantee threshold activation in each case. Current increase as a consequence of network transition to HCS is acquired as a time series. Rather than a continuous transition, we are able to distinguish the activation as a series of discrete current jumps. Comparison with the model shows that resistive switching in vital topological areas of the network produces this effect. When the fixed bias voltage is removed, network memorizes the HCS for varying periods of time. The memory state duration is random and varies between activation cycles. Stochastic dissolution of individual nanowire-nanowire junctions and the formation of multiple conductance pathways during the activation is combined with the model to explain this property. Finally, we devise a novel measuring scheme for the I–V activation cycle, which splits every voltage step change, while tracking the evolution of the network from a very low conductance state, towards activation and subsequent adaptation, leading to a final and very stable high conductance state. Analyzing the 1/f power-law spectrum distribution within these series, we disentangle the dynamical events under witch this optimized current transport state is automatically optimized by the network. This optimal state is nevertheless susceptible to random changes in the sub-threshold regime when vital topological sections of the network are compromised, but the network can quickly recover from such instabilities via an “avalanche-like” remapping of the preferred conductance pathways.

## Structural Properties of PVP-Ag Nanowire Networks

**Figure 1:**
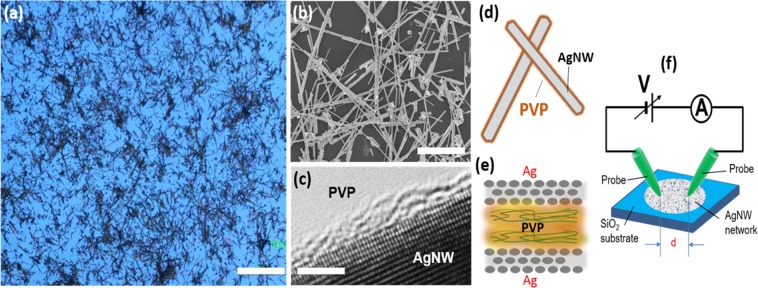
Morphological and structural properties of PVP-coated Ag nanowires and nanowire network. (**a**) Optical micrograph image of nanowire network layout after drop-cast deposition on a SiO_2_ substrate. (**b**) SEM image of nanowire interconnectivity in a selected area of the network. (**c**) HR-TEM image showing the atomic planes of the [100] facet of a Ag nanowire with the nanometric PVP layer embedded on the lateral surface of the nanowire. Figures (**d**,**e**) sketch the detail of the insulating junctions formed by the polymeric PVP layer between the Ag surfaces of overlapping nanowires. (**f**) Scheme of the measurement system. Two tungsten probes, separated by distance d = 500 μm, act as electrodes, contacting the nanowire network deposited on SiO_2_. The scale bars for figures (**a**–**c**) are 100 μm, 10 μm and 2 nm, respectively.

An optical micrograph image of a PVP-coated Ag nanowire network has been acquired after drop-cast deposition on a SiO_2_ substrate. As is evident in Fig. 1a, nanowires are dispersed with random orientations and their spatial distribution exhibits areas of denser connectivity interspersed with sparser regions. Higher-resolution scanning electron microscopy (SEM) (Fig. 1b) provides a more detailed view of the nanowire network, revealing a high-degree of interconnectivity between adjacent nanowires, including multiple overlaps. Small amounts of particle-like or irregular Ag impurities not removed by centrifugation are observed as well. The average diameter and length of nanowires was measured to be 360 ± 110 nm and 14 ± 5 μm, respectively. The network density is thus estimated to be 0.76 nanowires/μm^2^, which is controlled by the concentration of nanowires in solution prior to drop-casting onto the surface. Upon further inspection with high-resolution transmission electron microscopy (HR-TEM), a PVP layer enveloping the surface of a Ag nanowire is observed (Fig. 1c), which is primarily responsible for the preferential formation of nanowires in the final product^34^. The average thickness of the PVP layer measured by TEM was found to be 1.2 ± 0.5 nm. Owing to the insulating nature of PVP, the structure of the junction formed between two PVP-Ag nanowires is considered to be a resistive metal-insulator-metal junction, as sketched in Figs. 1d,1e. Resistive switching is observed when an electric field arises at the junction between two PVP-Ag nanowires as formation of a conductive filament through the PVP interlayer is promoted^37,39^. The highly-resistive nature of these junctions thus governs the electrical properties of the network prior to electroformation of conductive filaments across nanowire-nanowire junctions (referred to as junctions for the rest of the text).

## Electrical Activation of Current Transport in PVP-Ag Nanowire Networks

**Figure 2:**
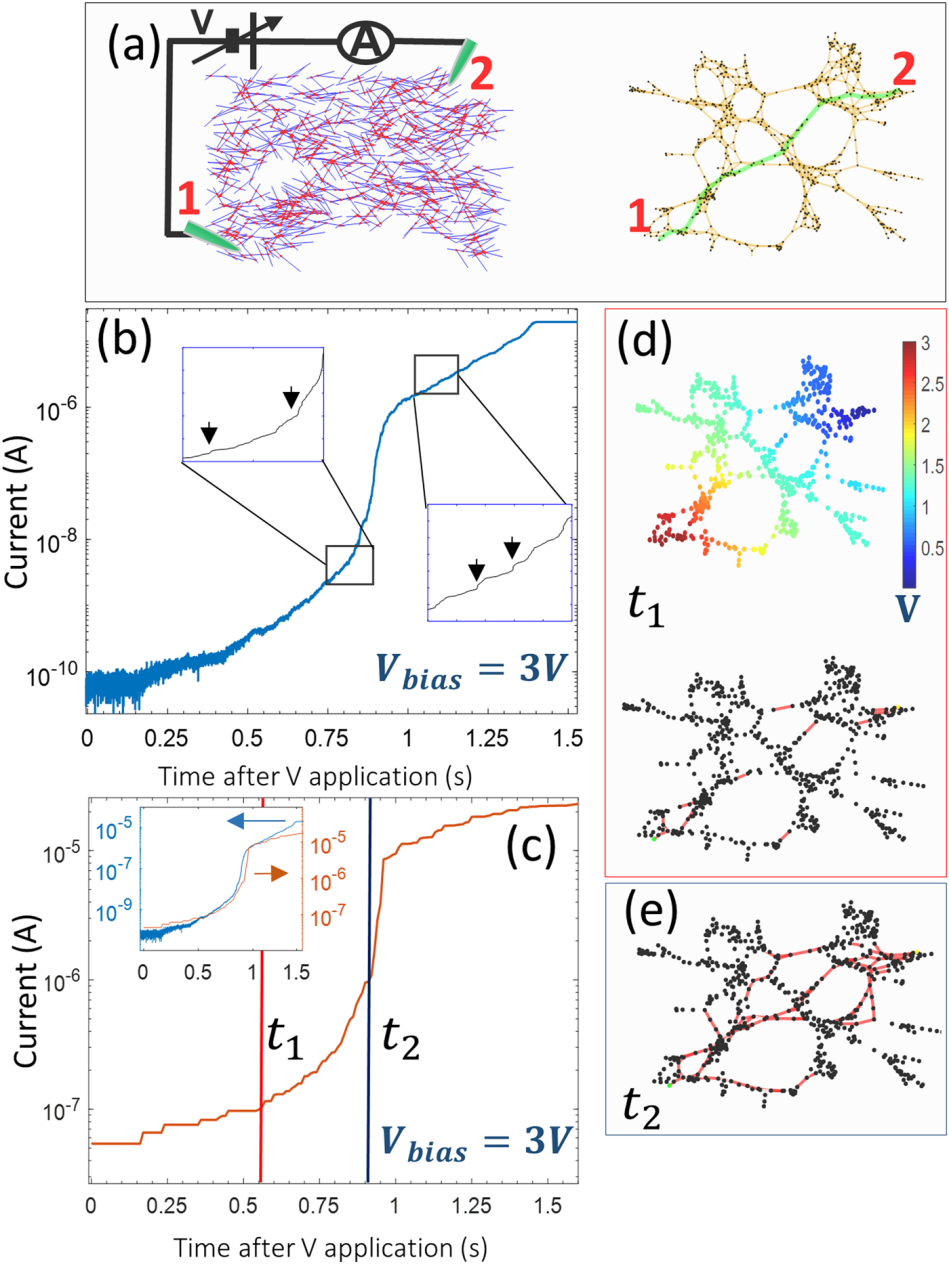
Dynamics of fixed-voltage superthreshold activation in PVP-Ag nanowire networks. (**a**) Left: Generated nanowire network displayed in a 100 × 100 µm^2^ two-dimensional grid, with two probes acting as electrodes. Blue lines represent nanowires and red dots represent junctions between two nanowires. Right: Graph representation of the network simulated on the left by using a force-directed layout. Edges are drawn in yellow and nodes in black. The edges drawn as green lines represent the topological shortest path between the two electrodes. Labels ‘1’ and ‘2’ mark the position of the electrodes. (**b**) Current vs. time measurement of network after applying a constant voltage of 3 V between electrodes. Two insets show a zoom-in section of the activation curve during two different time regions, highlighting particular features observed during the activation sequence. (**c**) Simulated time series of the network in (**a**), after applying a fixed interelectrode voltage of 3 V. Inset shows overplotted experimental (blue) and simulated (orange) activation curves. Two vertical lines, labeled as t1 (red) and t2 (blue) mark two selected time points during the activation sequence, at which the network state is represented in graph form in (**d**,**e**), respectively. (**d**) Voltage potential distribution colormap across the network nodes (nanowires, top) and the activated junctions (red edges) at t1. (**e**) Activated junctions (red edges) in the network at t2.

Critical, or threshold, activation is a known feature of metallic nanowires, atomic switches and nanoparticle networks. It results from the formation of low resistance conduction paths between electrodes^31^. In a typical I–V experiment, the threshold voltage depends on many factors, including network density, the nature of the individual junctions and the interelectrode distance. For a density of 0.76 nanowires/μm^2^, threshold voltage for a PVP-Ag nanowire network is relatively insensitive to variations in electrode separation and lies between 1.5–2 V^40^. We have tracked the evolution of electrical current on a time series at fixed electrode separation when a constant voltage bias larger than 2 V was applied to the network. Experimental setup is schematized in Fig. 2a, left. At both ends of the network, two probes are contacted and used as electrodes for biasing the network. Figure 2b shows the time evolution of current measured over the period under which networks undergoes electrical activation. The complete activation series resembles a sigmoidal shape. In the initial moments, current is negligibly small. An exponential increase is then observed, under which the conductance of the network increases more than two orders of magnitude in tenths of seconds. Just after activation occurs, a period of logarithmic increase in conductance is followed, before finally reaching the saturation range of the measurement system.

Detailed segments of the dynamics during activation have been plotted in the insets of Fig. 2b to outline characteristic noise fluctuations prior to and after activation. As can be seen in the insets, the activation curve does not increase monotonically but rather in a step-like manner. Neither the length of the plateaus nor their relative height follows a regular pattern. Irregular plateaus can be also observed just after network transitions to a high-conductance state. We used an atomic switch-like model for the nanowire-nanowire junction^21^ to simulate these features. The model accounts for the junction-to-network dynamics by combining electrical network solutions using Kirchhoff equations, with a filament growth model for the junctions which is functionally similar to the classical HP model of the memristor^4^. The filament length continuously changes between two junction states: open, in which filament length is less than the thickness of the PVP layer (high resistance), and closed, for which filament length is equal to the thickness of the PVP layer (low resistance). Junction resistance switches back and forth between these two regimes upon changes in the electrical current going through it. Details of the model can be found in the Supplementary Information.

The modelled network that was used to simulate activation series has been depicted in Fig. 2a on a two-dimensional grid, with similar density as the physical system. The graph of this network is also shown on Fig. 2a, right. The nanowire network layout closely recreates the inhomogeneous distribution of nanowires on the substrate produced by our experimental deposition method (Fig. 1a). The simulated current after applying a fixed bias voltage is displayed in Fig. 2c. The shape of the simulated activation curve closely resembles the experimentally measured curve (Fig. 2b). It exhibits a characteristic sigmoidal shape, with exponential activation at low conductance, followed by a critical activation period in which the network very rapidly switches from a low-conductance(LCS) to a high-conductance state(HCS).

In the simulation, the network current spans approximately two orders of magnitude. Due to the reduced size of the simulated network, conductance is higher in the simulated activation curve, as the bias voltage is similar in both cases. Qualitative agreement is nevertheless observed by comparing the overplotted experimental and simulated activation curves in the inset of Fig. 2c. Typically, the activation process in random networks is probed by acquiring I–V curves. In an activation time series, several network parameters such as density, local homogeneity and interprobe distance can influence the time it takes to activate the network whenever voltage is larger than the threshold, as well as the number and shape of plateaus. Furthermore, local junction activation in random discrete areas of the network due to random thermal fluctuations can preclude the formation of certain current pathways at the expense of others, as shown recently^41^. Despite this, the time series profile exhibits a universal sigmoidal shape. This universality was confirmed by activation curves acquired with different Ag-PVP nanowire networks (Fig. S1).

Further insight can be gained about fundamental physical processes occurring during activation by carefully inspecting the current and voltage distribution in the network represented as a graph, at two selected times (*t*_1_ and *t*_2_ from Fig. 2c). Figure 2a(right), 2d and 2e plot a graph representation of the Laplacian matrix of the electrical network. In this representation, nodes (black dots) represent nanowires and edges (red lines) represent individual nanowire-nanowire junctions. A force-directed graph layout was used to better visualize the underlying topological structure of the electrical circuit formed by the nanowire network^45^. In network regions where the local nanowire density is higher, the network is more highly interconnected. As a consequence, these areas can be regarded as a highly parallelized circuit in which current is more efficiently distributed through many individual open junctions. The local voltage variations between individual junctions are much smaller in highly interconnected regions. These regions of higher connectivity are bridged by few individual junctions connected in series, in which local voltage variations are larger (as shown in the equipotential voltage map of Fig. 2d, up). By comparing Figs. 1a and 2a with the graph, it is evident that the topological structure of the network electric circuit is comprised of a combination of parallel and series pathways between both electrodes (nodes labelled ‘1’ and ‘2’). The green line in the graph of Fig. 2a (right) highlights the edges along the shortest-path between both electrodes. A breadth-first search algorithm, assuming equal weight in all edges, was used to compute this path.

In the initial state of the network, with all junctions opened, the inhomogeneous density of the nanowire deposition produces an irregular voltage distribution. Figure 2d, down and 2e show the distribution of opened junctions at *t*_1_ and *t*_2_. For clarity, only the edges for which the nanowire-nanowire junctions are switched on (low resistance) are drawn as red lines, leaving the other edges blank. Larger voltage drops appear in the more resistive areas of the network, as evident from Fig. 2d, which implies that these junctions are the first to switch to a closed state. Junctions closing near the electrode and in between areas of higher density will result in discrete jumps in current, as the effective resistance of the network is reduced. The exponential increase in current just before transitioning to a high-conductance regime occurs as junctions in denser areas of the network switch in an avalanche-process, bridging the low resistance pathway. Figure 2e outlines the state of the network just before activation, in which a single pathway of closed junctions forms a connected channel. Subsequent discrete current plateaus observed after activation (Fig. 2b (inset) and 2c) arise as parallel low resistance channels are formed in the network. When all available pathways are active, the network reaches a final conductance state. Analyzing the current distribution per junction in the modelled network, we found that only 25% of the junctions carry more than 1 µA (Fig. S2).

## Short-term Memory and Stochastic Breakdown

**Figure 3:**
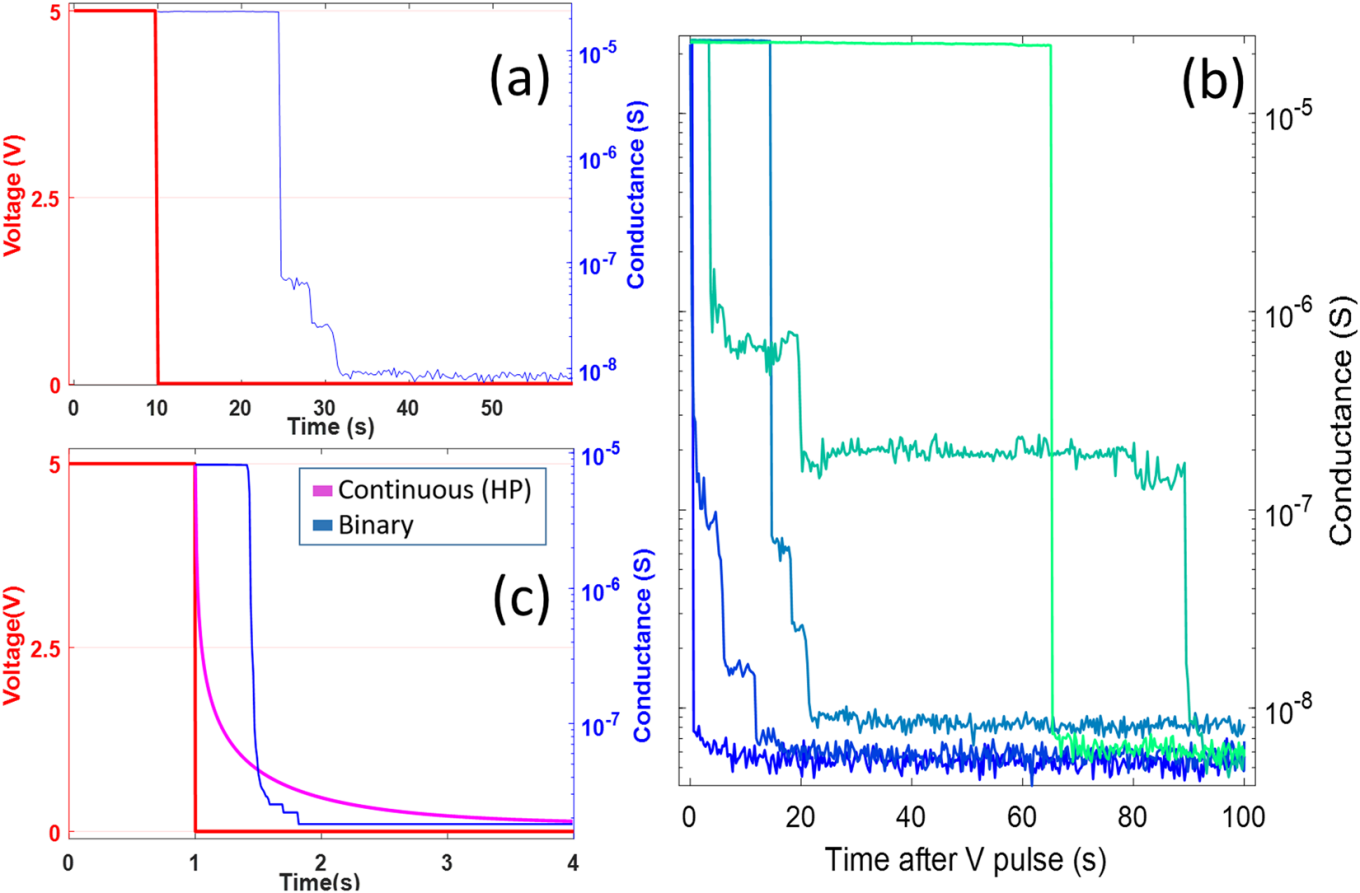
Probing memory retention dynamics of a PVP-Ag network. (**a**) Changes in network conductance after applying a square pulse between two electrodes. Red line: a 5 V voltage pulse profile was used to activate the network and 10 mV to read current during network deactivation. Blue line: network conductance response time profile. (**b**) Typical deactivation profiles acquired during 100 s after applying square pulses, same parameters as in (**a**). (**c**) Simulated data using the same pulse profile as in (**a**) for a nanowire network generated on a 100 × 100 µm^2^ two-dimensional grid. Reading voltage was 0.1 mV in the simulated network, to account for the reduced size of the grid. The decay dynamics of two different junction resistance models, Continuous (or HP), pink line, and discrete (or Binary), blue line, is overlaid for comparison.

The interplay between adaptability, structural inhomogeneities and current transport across electrical nanowire networks gives rise to non-linear temporal dynamics in the activated network after the voltage is removed, as shown in Fig. 3. Here, the nanowire network was electrically stimulated by a square pulse signal with fixed amplitude (5 V) and duration (10 seconds). This setting was chosen to ensure complete activation of the network in each series. After each activation, the voltage bias was reduced to 10 mV and current was acquired during the following 100 seconds. The network’s conductance response post-stimulus (Fig. 3a) reveals a complex deactivation cycle, non-monotonically decreasing and abruptly transitioning between plateaus with different conductances at irregular time intervals. This is in stark contrast to a fixed resistor network, which would maintain the same conductance, but almost immediately transition to proportionally lower currents when reducing voltage. The observed nanowire network behavior is instead consistent with a short-term or retention memory that can be attributed to the collective response of the individual junctions. Current transport is necessary to stabilize each junction^46^, but can then remain in a closed state even when the bias is reduced to a value much lower than the activation voltage (or sub-threshold level). Activation-deactivation cycles were repeatedly applied to the network and different deactivation traces were recorded. A selection of typical traces is shown in Fig. 3b, which shows that in the first 100 seconds after the pulse is applied, the network deactivates and transitions to a low conductance state. Deactivation traces regularly exhibit similar characteristic patterns as in Fig. 3a, with sharp drops in conductance followed by irregular plateaus. In many traces, the network abruptly loses its conductance after voltage removal. In other cases, HCS is retained for long periods of time, up to 60 s (bright green line), while in intermediate cases network loses the HCS in step-wise irregular plateaus at intermediate conductance states. We have analyzed the distribution of times between switching events (drops in conductance) to outline the random nature of these (Fig. S3).

This activation-deactivation measurement sequence was simulated to recreate deactivation traces. Figure 3c shows the simulated network conductance response using the same network parameters and geometry as in Fig. 2a. The shape of the simulated response (blue line) closely resembles the experimentally measured response (Fig. 3a), showing similar features: high-conductance state memory retention and abrupt conductance drops followed by irregular plateaus. In the model, resistance between junctions is controlled by an internal state variable, the length of the Ag filament. This length grows with electrical current, which promotes the development of the junction, and shrinks through an exponential decay term. This term is used to reproduce the inherent instability of atomic switches at room temperature^28,46^. The physical mechanism whereby Ag filaments decay could be related to spontaneous clustering of the Ag filament whenever voltage difference is absent, in order to minimize surface energy of the filaments^47^. Thus, we have included a dissolution threshold in the filament length. If the filament length falls below this dissolution threshold, individual junctions immediately switch back to an open state. When the voltage applied to the activated network drops below a certain level, the junctions will become unstable and susceptible to breakage or shrinking. Although junctions near the electrodes or spanning denser regions of the network show stronger resilience during voltage reduction, they eventually become unstable and break, producing the distinctive sharp transitions in conductance, as highly interconnected areas are successively disconnected within the network. As can be noted by comparing Fig. 3a,c, the agreement between the simulation and the experiment is only qualitative. The typical decay times for the experimental curves are unpredictable and about 10 times longer than the simulated one (Fig. 3b), while the model, even with this dissolution threshold approximation, is deterministic, and as such, the decay time is determined by the decay term used in the simulation, which was 1 s. To further clarify the validity of this approximation, in qualitative terms, we have included in Fig. 3c the decay curve (pink) using a continuous model for the resistance evolution when the filament length shrinks^21^. In this model, there are no dissolution threshold below which junction resistance drops due to filament breakage. The exponential decay curve represents an average decay of the ensemble of individual junctions participating in current transport, which produces a smooth reduction in the equivalent resistance of the network. Evidently, this junction resistance model is not able to capture the underlying transport hierarchy existing within the network pathways, and neglects the underlying *potentiation-in-decay* mechanism responsible for the dynamical behavior of the network. The resistance model with dissolution threshold (or binary) combined with the observed decay traces (Fig. 3b), on the other hand, suggests that the different deactivation responses can be attributed to two effects: the stochastic nature of the filament-breaking junctions^28,46^ and the potentiation of the backbone current pathways when very low voltage is present. This combined effect gives rise to the creation of multiple meta-stable states of the network, appearing as irregular plateaus at different conductances with different lifetimes. Thus, this mediation of stochastic thermal junction breakdown not only probes the existence of several conduction channels actively participating in current transport, but also produces a continuous and adaptive readjustment of the preferred conductance pathways when the network loses conductance. The fact that we observe conductance plateaus signals the extraordinary fault-tolerance of the network, as the backbone of the network must carry more current to maintain the same conductance level.

## Transport Optimization and 1/f-Power Law Dynamics

**Figure 4:**
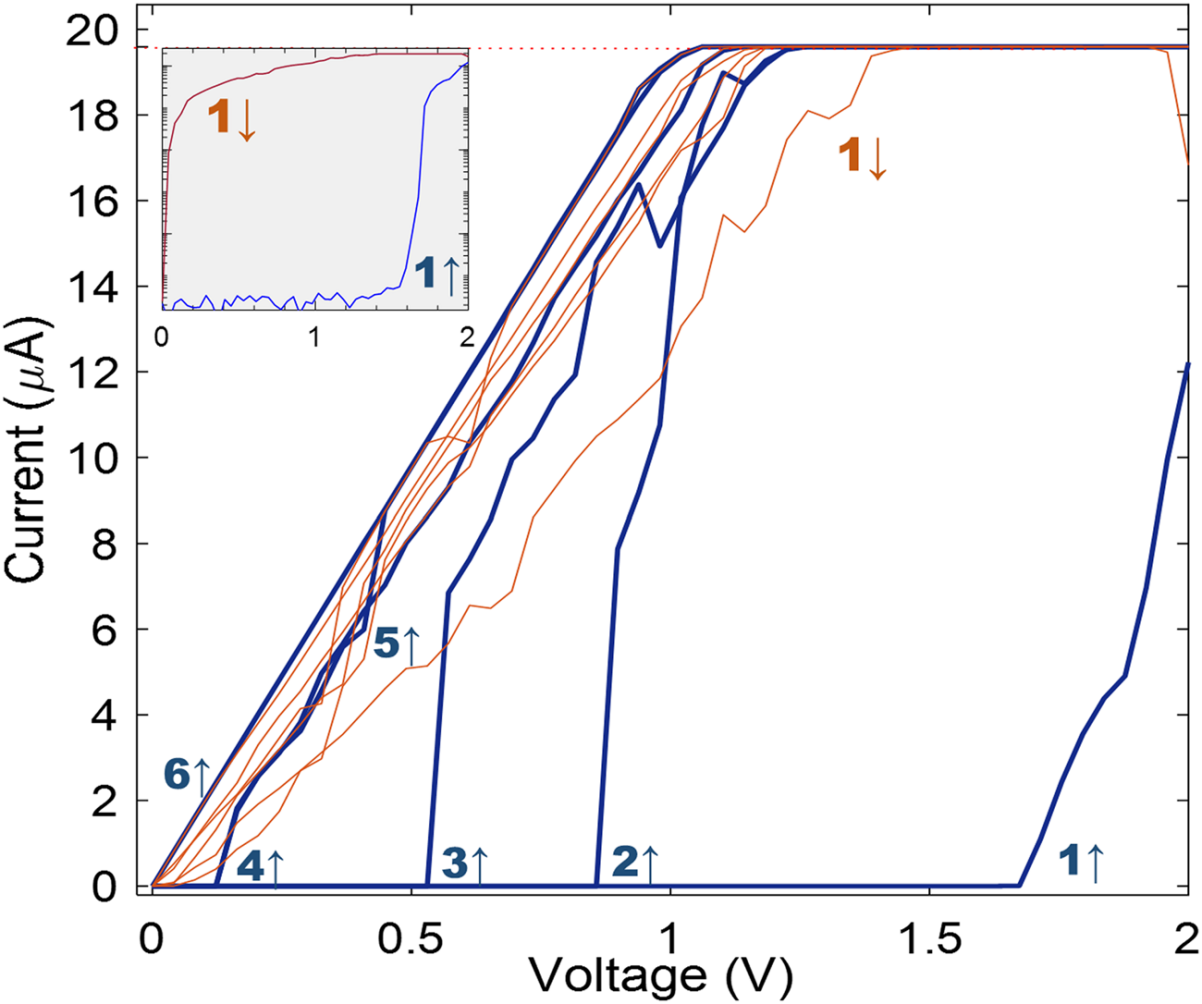
Evolution of PVP-Ag nanowire network connectivity under consecutive I–V cycles. Six up-down cycles are shown in the figure. Upward voltage ramps are drawn as blue lines, and labeled with the cycle number and an arrow pointing upward. Downward voltage ramps are drawn as orange lines; for clarity, only the first cycle is labeled. The inset shows the first I–V cycle in logarithmic scale.

Figure 4 shows the nanowire network evolution upon applying consecutive I–V cycles. The first upward ramp (blue line, labelled ‘1’) shows the network transitioning from a disconnected or low conductance regime, which produces no detectable current, up to the exponential activation of the network when the voltage is above activation threshold (cf. Fig. 2). The voltage is immediately ramped down and in the first downward cycle, the network current reduces approximately linearly. The fluctuations observed in the downward trace can indicate a low degree of connectivity in the network. On the next upward cycle (blue line, labelled ‘2’), sharp activation occurs again but at a lower threshold voltage. The tendency is repeated in the two following upward cycles. As the I–V waveform was 10 Hz, the time between consecutive ramps is less than the typical decay times (short-term memory) observed in Fig. 3, and as memory is retained in successive I–V cycles, upward voltage sweeps strengthen the network connectivity until the final cycle (‘6’), in which both upward and downward curves show a completely stable linear profile, indicative of an ohmic system. This state is stable as long as I–V cycles are driven in the same positive voltage range and compliance current.

**Figure 5:**
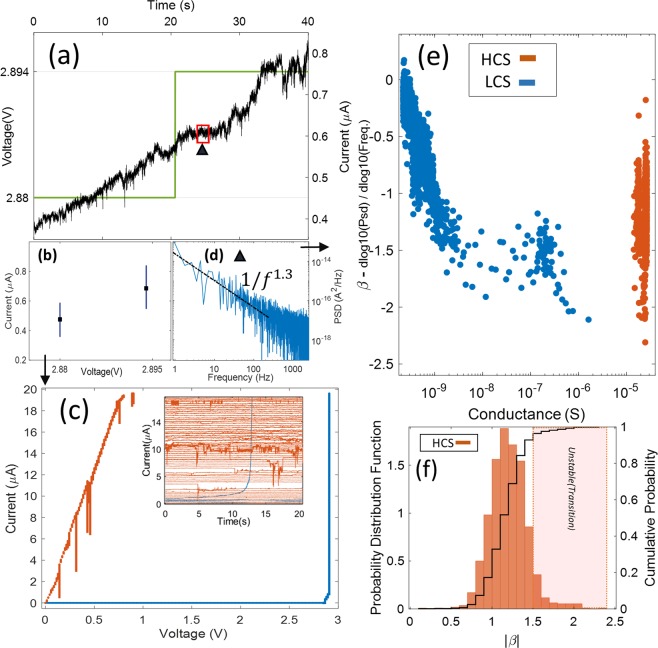
PVP-Ag nanowire network dynamics under a sliced-time I–V curve from 0 to 3 V. (**a**) Current time series acquired during an upward voltage step ramp. Voltage steps up by 14 mV and current is acquired for each 20 s window of fixed voltage on either side of the step. (**b**) Current measured during each 20 s duration of fixed voltage on either side of the step in (**a**) is plotted as a vertical line (black dot indicates mean value). (**c**) Typical activation curve obtained by combining current measurements for all upward (blue) and downward (orange) voltages. The inset shows current vs. time series acquired for each upward (blue) and downward (orange) voltages (for clarity, the starting time for all series has been shifted to the origin. (**d**) Power Spectral Density (PSD) of the current time series in (**a**) computed for the 1 s window marked by an upward triangle. The black line shows the power-law best fit (note the log-log scale),with exponent ***β*** = −1.3. (**e**) Changes in the PSD power exponent with network conductance calculated for 1 s time windows in all the measured current-voltage data. Points corresponding to the upward voltage ramp (low conductance state, LCS) are colored blue, orange points denote downward voltage ramp (high conductance state, HCS). (**f**) Probability distribution of PSD power exponent absolute values for the high conductance state network, overplotted with the cumulative probability.

The time series profiles in Figs 2 and 3 reveal underlying features of the switching across the network when voltage is applied continuously or in discrete pulses, while the IV cycle in Fig. 4 contains information about the evolution of the network toward a preferred or definitive high conductance state. To gain further insight into the interplay between the switching dynamics and the characteristic activation cycle, we devised a new measurement scheme to combine the time series and I–V acquisitions. In each voltage step of the I–V cycle, current is acquired for a given amount of time (20 s), the time series for each step are analyzed independently instead of averaged. The current time series acquired during two consecutive voltage steps during the upward ramp is shown in Fig. 5a. The network was in a voltage close to the critical activation. The current increases approximately linearly in this range as voltage is ramped up. The current fluctuations are in the order of tenths of μA. After acquisition, we reconstruct the I–V curve by plotting every voltage step during the upward (and downward) cycles against the span of current measured during that fixed amount of time. Thus, current time series becomes a vertical line when plotted as current versus voltage, as shown in Fig. 5b. This is in contrast to the conventional averaging method that results in a single point for every voltage (black dots in Fig. 5b). This analysis method allows us to observe not only the current vs. voltage distribution of the I–V cycle, but also the relative stability of the network against voltage variations (as accounted for by longer (shorter) lines when network current fluctuates more (less) for a given voltage). The resulting curve is displayed in Fig. 5c, in which the lines corresponding to the upward (downward) cycle are colored blue (orange). The activation cycle is similar to that seen in the I–V curves in Fig. 4, but with noticeable differences. First, the onset of a high conductance regime occurs at a slightly larger voltage. This implies that the activation dynamics of the network is altered when following this measurement scheme. The duration of every voltage step (20 s) is longer than the typical time to deactivate a network (Fig. 3b), and the height of each voltage step (14 mV) is small compared to the threshold voltage (~2 V). The increase in fluctuations just before network activation (Fig. 5a) suggests that there is an increased competition between thermal junction breakdown and junction formation. Additionally, the network topological layout and nanowire density inhomogeneities can also affect the activation sequence.

We computed the power spectral density (PSD) of every current time series. Similarly to performing a Short-Time Fourier Transform (STFT), time series are subdivided in fixed intervals (one second each in this case). Spectral analysis is computed on each of these separately allowing to disentangle different dynamical regimes occurring during the I–V cycle. Figure 5d shows a PSD computed from the time segment shown by a red box in Fig. 5a. Changes in the characteristic exponent of the resulting power-law dependence reflect dynamical changes in the network. The exponent, also termed β^48^ is the slope of the line of best fit to the PSD in a log-log plot. This non-zero slope arises as a consequence of correlations in the current fluctuations of different magnitude caused by the switching dynamics^18^. Figure 5e shows how β changes during the complete I–V cycle, as a function of the average conductance during each time frame.

Two different network conductance states are apparent upon inspection: the low conductance state regime (LCS, blue dots), which spans a range 10^−10^–10^−6^ S, and the high conductance state (HCS, orange dots), above 10^−5^ S. The LCS and HCS regimes coincide with voltages corresponding to the upward and downward ramps, respectively. In the LCS regime, all β values are scattered between 0 and −2, and exhibit a steep linear increase up to ~10^−9^ S, followed by an approximately constant but disperse distribution of values around −1.5 until the activation voltage, at which conductance is 10^−6^ S. The HCS regime shows a much different distribution, with β values between −0.5 and −2.2 centered in a narrow conductance band near 2–3 × 10^−5^ S. The magnitude of the power-law exponent β is regularly used in the analysis of different dynamical processes. A magnitude of the exponent close to 1 is often referred to as “1/f noise”, and has been found to appear in many dynamical processes. Despite its ubiquity, the origin of such behavior is still a matter of debate^48^, but may be related to the system in a state of self-organized criticality^49^, in which the interaction between low-frequency and high-frequency processes is hierarchically organized in a scale-free, coherent manner. This self-organized state has been observed in neural dynamics^43,50^. Processes with β exponent close to 2 are characteristic of Brownian systems^51^.

When the network is fully inactive (<10^−9^S conductance), it can be considered as a system with very large equivalent resistance, as current falls in the noise level of the amplificator. The approximately flat PSD is thus a consequence of thermal (white) noise induced by the sub-nA current through the amplification system, and any physical process occurring in the network are masked by a larger noise amplitude. As voltage increases, junctions start to close, diminishing the equivalent resistance in discrete steps (Fig. 2c), and different regions of the network becoming connected. In Fig. 5e, the linear dependence in the β values at |β| > 0.5 reflects the degree to which the current fluctuations produced by discrete junction switching overcomes the amplificator’s noise. Beyond the nA regime, the signal-to-noise ratio is sufficiently high that the switching dynamics of the network is the dominating factor in the entire spectra (Fig. 5d). As discussed before, in this pre-activation regime, the avalanche-like junction opening that produces sharp and strong transitions in the I–V cycle is counterbalanced by thermal desorption of single junctions due to large acquisition times for every point, and β values are distributed around -1.5. Just before activation (rightmost 10 blue circles in Fig. 5e), the much larger and irregular current fluctuations (rightmost blue line in Fig. 5c) are distinguished by β values close to −2, after which a sharp transition to the HCS occurs (red points in Fig. 5e).

Figure 5f shows the corresponding probability distribution of |β|values when the network is in the HCS. The β distribution is centered closed to -1(average value of −1.2.) showing an asymmetric tail at |β| > 1.5. As is evident from comparison of the corresponding data (red dots) in the downward ramp of Figs. 5c,e, the network HCS is very stable against current fluctuations. This resilience against even the strongest current fluctuations (longest vertical lines in the downward ramp data in Fig. 5c) can be attributed to its ability to adapt and redistribute signal power to lower-frequency collective modes. We ascribe the tail of the β distribution observed in Fig. 5f to these unstable states, manifested as sharp, irregular and random fluctuations. In this particular example, the network is relatively stable during the whole downward cycle, and we estimate from the cumulative distribution in Fig. 5f that the probability to enter this unstable regime is 3% (shaded box in Fig. 5f). By repeating the experiment for different networks, the transition to a definitive or stable high-conductance state is also observed, but this state is sometimes more unstable so the whole distribution is shifted towards larger β values, as can be seen in the Supplementary Information (Fig. S4).

## Discussion

It has been shown in previous sections that PVP-Ag nanowire networks evolve towards a definitive state of conductance after becoming fully activated, either by means of repetitive I–V cycles or applying super-threshold voltage over longer times. In the activated network, one or many pathways transport electrical currents. The modelling results (Fig. 2a,d,e) suggest that the pathway that opens first is the shortest-distance topological path between the electrodes. However, the model assumes a homogeneous initial distribution of junction resistance, which may not be the case in the real system, which has a larger interelectrode distance, as well as non-ideal contact geometry between nanowires and between nanowires and electrodes. Additionally, the self-assembled network may exhibit larger homogeneity variations that are not accounted for in the model. This implies that filament formation at the level of individual junctions might not always lead to an efficient activation sequence. For example if one junction in a less optimized pathway change its resistance state first, it may promote the activation of junctions in its immediate vicinity, thus giving rise to a winner-takes-all phenomenon, as recently observed by Manning and colleagues^41^ in a smaller PVP-Ag network. In any case, once a pathway is active, the network will keep further opening parallel pathways in order to optimize the conductance, or else, minimize power consumption, in order to comply with the laws of conservation of electrical current in a resistor network

**Figure 6:**
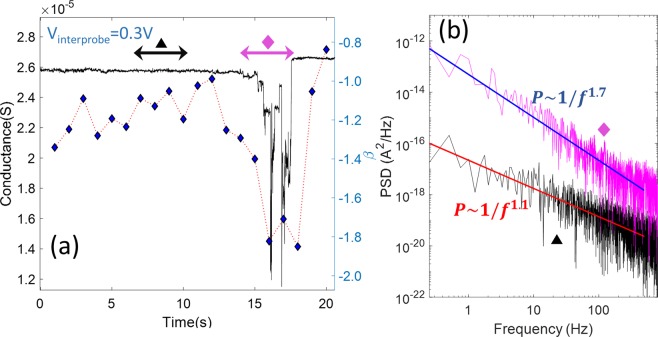
Reconfiguration dynamics in PVP-Ag nanowire networks. (**a**) Conductance time series selected at the 0.31 V step during the downward ramp of the IV cycle, with an overplot of the slope (β) of the power-law fit to the PSD computed for 1 s intervals (blue diamonds). The different spectral distributions of power in the self-organized dynamical regime (indicated by a black triangle) with the unstable regime (indicated by a pink diamond) are compared in (**b**). In each case, a linear fit is overplotted on the PSD.

Previous studies^36^ have shown the stability of active networks to last extremely long times as long as the energy pumped from a voltage or current source is high enough. Thus, once in the ohmic regime, a network can remain in this state unless, by means of a higher voltage or current injection, other physical phenomena such as joule heating or electro-migration breaks the individual junctions, thereby resetting them^36,52^. In our study, we have inspected the dynamics of the active network not only near the threshold voltage, but also in the sub-threshold regime. In this regime, the stability of the networks is still very high, but as the current through individual junctions decreases, the balance between junction formation by means of current injection and junction dissolution triggered by stochastic fluctuations is reduced. Decreasing the voltage in a linear sweep while acquiring time series facilitates the inspection of the dynamical events, signaling network instability by single junction breakdowns. This is assessed by analysis of the PSD power-law exponent (β) distributions in Figs. 5d,e. The β distribution in Fig. 5e shows that most of the magnitudes are centered around 1, with a small tail of values rounding up to a magnitude of 2. Typically, a 1/f power-law spectrum is indicative of a system in which scale-free dynamics is at play. To better understand how the network changes its dynamical behavior, we dissected the downward voltage ramp of the I–V cycle of Fig. 5c and examined the conductance time series at a voltage in which this dynamical balance was evident. Figure 6a shows that the network maintains a very stable (small fluctuations) conductance near 2.6 × 10^−5^ S up to approximately 15 s, after which it suddenly becomes unstable, exhibiting sharp and irregular conductance drops interspersed with short plateaus of unequal duration and magnitude, as well as large spikes. This unstable regime last for 3 s, after which the network automatically recovers the previous stable state, only this time with a slightly higher conductance. The level of fluctuations in this new stable regime appears to be slightly lower. Figure 6a also shows β values calculated at every one second interval in the conductance time series. This analysis reveals that when the network is in a dynamical regime that is stable against fluctuations, β values are closer to 1, whereas they approach 2 when the network succumbs to instability. Figure 6b shows PSDs calculated for a stable and unstable region in the conductance time series in Fig. 6a, indicated by the black triangle and pink diamond, respectively. The PSDs clearly distinguish the different dynamical regimes: the PSD is steeper in the unstable regime due to the stronger low-frequency fluctuations. Upon further inspection of Fig. 6a, with reference to the full distribution of sliced time series in Fig. 5c, it is evident that the onset of the unstable regime is not triggered by the negative voltage sweep, nor it is related to changes in the network appearing immediately after one voltage step is performed in the downward ramp, but instead appears randomly and unpredictably, and with an irregular duration. The unstable regime ends when the network recovers a stable conductance state, as signaled by reduced noise fluctuations. Therefore, the network not only adapt its conductive pathways to the nanowire junction topology whenever energy is supplied either from current or voltage sources, but can also adapt dynamically to random changes in the network, reconfiguring the underlying connectivity to find a new optimal pathway.

Few nanowire network models have addressed stochasticity in the sub-threshold regime^21^. Our modelling of the activation and deactivation cycles (cf. Figs 2 and 3) provides new insights into the process leading to these changes in network. When the applied voltage is much lower than the threshold voltage, the transport hierarchy is revealed through the different irregular decay curves (Fig. 3b), attributed to stochastic junction breakdown. In reducing the voltage from threshold to sub-threshold level in small steps and long waiting times, we were able to track the transition from the stable state down to the regime in which random fluctuations have a larger influence on the dynamics. Whenever β is close to 1, individual junctions within clusters of high network connectivity are continuously switching off and on, with three different dynamical process (potentiation, inhibition and random breakdown) interacting and evolving in a self-organized way such that the connectivity backbone of the network is not compromised. Therefore, a junction that breaks in a cluster of high connectivity is propagated as a small change in conductance, and, depending on the local topology of these clusters, induces the breakdown of neighboring junctions, as the current in them is instantaneously reduced. But at the same time, when these secondary junctions are broken, the backbone of the network needs to carry more current to maintain conductance, so if a region is locally connectivity-depleted, another region will have to sustain a larger current, and thus, will potentiate the switching-on of junctions over that area. However, the more junctions are switched on, the higher the probability of breaking junctions in these newly connected areas. In this way, self-organized feedback produces the scale-free power-law distribution, as fast individual junction switching leads to changes in conductance for clusters of junctions over a slower time scale, and changes in cluster conductance produce changes in network pathway connectivity on an even slower time scale. This hierarchy is responsible for the characteristic 1/f spectrum in the PSD. From this interpretation, we can infer that the steepening of the power-law spectrum associated with abrupt changes in conductance (cf. Fig. 6a) occurs when a critical junction (one bridging areas of larger connectivity) breaks down, and splits a network pathway, reducing the total network conductance (by as much as half in the ideal case of two equivalent parallel pathways). Then, self-organized switching is disrupted by larger junction connectivity-depletion, and the connectivity of the network is spontaneously remapped until the broken pathway, or another parallel pathway, is switched back on. This remapping (or reconfiguring) process produces more low-frequency power in the PSD, as clusters of junctions or secondary pathways collectively redistribute connections, which occurs on longer time scales (lower frequencies) than the higher frequency switching fluctuations of individual junctions. When the network transitions back on to a highly conductive state, the conductance is slightly different, implying this complex dynamical process produces long-lasting changes in the backbone connectivity of the network. We can draw a parallel with long-term retention memory in the brain, which requires not only synaptic potentiation in neural connectivity but also persistent structural reconfigurations in connectivity, referred to as plasticity^53^.

Further insight into the relation between dynamics and connectivity can be gleaned by encompassing the neuromorphic nanowire network within a broader class of systems, optimal transport networks. In general, these are networks that can be represented by a weighted graph (directed or undirected), with edges representing the conductance (or resistance) between the different parts of the network, and whose solution can be obtained by solving a generalized version of Kirchhoff’s equations, that is, flow and mass conservation. Examples of such systems vary from artificial systems, such as power or water supply networks, to natural systems, e.g. vascular networks in plants or animals^54^. The optimality condition, though not always solvable, is computed by considering that transport is optimized whenever the connectivity map (weight between edges) minimizes power consumption. For example, for a fixed resistor network, current is hierarchically and homogeneously distributed in proportion to the equivalent resistance of the different parallel plus series pathways. In contrast, in vascular systems, which adapt flow to pressure differences^54^, dissipation is minimized by concentrating flow on the edges with larger conductance, thus transport is regulated by the formation of tree-like structures^55,56^. Neuromorphic networks clearly show this adaptive behavior, with the system evolving to create few, large connectivity pathways rather than many different pathways, as our activation curve and other recent research shows^41^. However, once the network is connected, parallel pathways, most likely in the form of closed loops or circuits that assist or reinforce the main pathway also appear. This further reduces global power dissipation. Interestingly, research on optimal transport structures suggests that the combination of a fixed unique backbone for transport with different recursively nested loops or cycles is the preferred connectivity map for adaptive networks, and appears once variations in the transport dynamics are introduced in the form of defects or fluctuating currents in the junctions, sources or sinks^55–58^.

Finally, it is worth considering whether the network dynamics observed in our neuromorphic nanowire system are characteristic of biological neural systems. In particular, we always observe a continuously fluctuating state, as if the system needs to be constantly oscillating, searching the phase space for a solution that guarantees long-term stability. It has been suggested that this *edge-of-chaos* state could be not only an optimal state of the brain, but a necessary condition for consciousness to exist^43,59^. However, although neural dynamics research has tended to focus on wave-like collective oscillations (e.g. in EEG signal acquisitions), other time series analyses continue to be developed^50,60,61^. Power-law PSD spectra, such as that observed in our nanowire networks, are indeed also observed in neural dynamics (e.g. neuronal avalanche size distribution), although whether these spectra can be interpreted as evidence for the brain operating at or near criticality remains a contentious issue^62^. Changes in power-law slopes, as observed in our nanowire network system, have also been observed in brain measurement data, where a subject performed simple tasks^60^. In our neuromorphic nanowire network, large changes in the power-law dynamics arise as random fluctuations break critical junctions. Introducing a control mechanism to disrupt selected regions of the network could force this remapping mechanism, that ultimately will allow us to harness the natural nonlinear feedback dynamics, thus navigating the large solution space of the network to solving different optimization or recognition tasks.

## Conclusions

We have analyzed the emergent dynamical properties of a neuromorphic system formed by polymer coated silver nanowires that self-assemble into a network with resistive switch electrical junctions. Our electrical measurements, performed with a large interprobe distance across the network, reproduced two important current-voltage properties observed in similar neuromorphic systems: critical activation at a threshold voltage; and evolution towards an optimal conductance state under successive cycles. We devised a new measurement scheme that unveiled even richer and more complex network dynamics arising from multi-scale interactions of the switch junctions, including: (i) a collective memory response in the sub-threshold voltage regime; (ii) distinct low and high network conductance states distinguished by different power-law fluctuation scalings; and (iii) reconfiguration dynamics, similar to synaptic plasticity in the brain, enabling resilience and adaptation. In particular, we found that the emergent temporal correlations arising in the active network under changing voltages exhibit a 1/f power-law spectrum, which is a consequence of fundamental changes in the connectivity map of the network and is primarily mediated by stochastic fluctuations at the individual junction scale that lead to hierarchical collective dynamics, from the scale of junction clusters to the whole-of-network scale. We envisage that by accessing the network’s states through its spectral signatures, we may not only gain a better understanding of its near-criticality dynamics, but may also discover new strategies for controlling training and learning for reservoir computing applications.

## Methods

### Synthesis

PVP-Ag nanowires were synthesized by the well established ‘polyol process’^33,34^, using 1,2-propyleneglycol(PG) as oxidizing agent for silver nitrate (AgNO_3_). Details of our procedure can be found elsewhere^63^. After synthesis, PVP-Ag nanowires were purified by centrifugation in water and dissolved in isopropyl-alcohol (IPA).

### Deposition

The PVP-Ag networks were prepared by drop-casting nanowires in solution onto SiO_2_ substrates. Density was sequentially controlled with an optical microscope. The network samples were further dried in a vacuum desiccator.

### Electrical characterization and analysis

Electrical signals were measured using two electrochemically etched tungsten probes as electrodes to make nanometer-scale contact with the network. The application of bias voltages between the electrodes allowed us to detect electrical currents flowing through the network. which were amplified with a custom-made current-voltage amplifier-converter. The probe positions were individually controlled by piezoelectric actuators in conjunction with an in-house-built positional control system^64^. Current was converted to voltage and amplified with an in-house-built amplification system, which was connected to a NI-USB6212 USB multifunction I/O device (National Instruments), for further digitization of the signal. I/O control and storage of data was managed by custom software developed in-house using Labview 2017 (National Instruments). Data was collected, analyzed and processed using MatLab R2017b (Mathworks).

## Supplementary information

Supplementary Information

## References

[1] Roth G, Dicke U (2005). Evolution of the brain and intelligence. Trends Cogn. Sci..

[2] Gentsch, P. AI Eats the World. In *AI in Marketing, Sales and Service* 3–9, 10.1007/978-3-319-89957-2_1 (Springer International Publishing, 2019).

[3] Cesar, R. M. & da Fontoura Costa, L. *An introduction to neural networks*. *Neurocomputing* **14**, (CRC PRESS, 1997).

[4] Strukov DB, Snider GS, Stewart DR, Williams RS (2008). The missing memristor found. Nature.

[5] Johnsen GK (2012). An introduction to the memristor - a valuable circuit element in bioelectricity and bioimpedance. J. Electr. Bioimpedance.

[6] Jo SH (2010). Nanoscale Memristor Device as Synapse in Neuromorphic Systems. Nano Lett..

[7] Cai, W. & Tetzlaff, R. Synapse as a Memristor. In *Memristor Networks* 113–128, 10.1007/978-3-319-02630-5_7 (Springer International Publishing, 2014).

[8] Kuzum D, Yu S, Philip Wong H-S (2013). Synaptic electronics: materials, devices and applications. Nanotechnology.

[9] Indiveri G, Linares-Barranco B, Legenstein R, Deligeorgis G, Prodromakis T (2013). Integration of nanoscale memristor synapses in neuromorphic computing architectures. Nanotechnology.

[10] Prezioso M (2015). Training and operation of an integrated neuromorphic network based on metal-oxide memristors. Nature.

[11] Kim KH (2012). A functional hybrid memristor crossbar-array/CMOS system for data storage and neuromorphic applications. Nano Lett..

[12] Bayat FM (2018). Implementation of multilayer perceptron network with highly uniform passive memristive crossbar circuits. Nat. Commun..

[13] Milano, G., Porro, S., Valov, I. & Ricciardi, C. Recent Developments and Perspectives for Memristive Devices Based on Metal Oxide Nanowires. *Adv. Electron. Mater*. 1800909, 10.1002/aelm.201800909 (2019).

[14] Terabe K, Hasegawa T, Nakayama T, Aono M (2005). Quantized conductance atomic switch. Nature.

[15] Waser R, Aono M (2007). Nanoionics-based Resistive Switching Memories. Nat. Mater..

[16] Ohno T (2011). Short-term plasticity and long-term potentiation mimicked in single inorganic synapses. Nat. Mater..

[17] Hasegawa, B. T. *et al*. Learning Abilities Achieved by a Single Solid-State Atomic Switch. 1831–1834, 10.1002/adma.200903680 (2010). 10.1002/adma.200903680 20512956

[18] Avizienis AV (2012). Neuromorphic Atomic Switch Networks. PLoS One.

[19] Stieg AZ (2012). Emergent Criticality in Complex Turing B-Type Atomic Switch Networks. Adv. Mater..

[20] Demis EC (2015). Atomic switch networks—nanoarchitectonic design of a complex system for natural computing. Nanotechnology.

[21] Sillin HO (2013). A theoretical and experimental study of neuromorphic atomic switch networks for reservoir computing. Nanotechnology.

[22] Scharnhorst, K., Woods, W., Teuscher, C., Stieg, A. & Gimzewski, J. Non-Temporal logic performance of an atomic switch network. *Proc. IEEE/ACM Int. Symp. Nanoscale Archit. NANOARCH 2017* 133–138, 10.1109/NANOARCH.2017.8053728 (2017).

[23] Bose SK (2015). Evolution of a designless nanoparticle network into reconfigurable Boolean logic. Nat. Nanotechnol..

[24] Minnai, C., Bellacicca, A., Brown, S. A. & Milani, P. Facile fabrication of complex networks of memristive devices. *Sci. Rep*. **7**, (2017). 10.1038/s41598-017-08244-y PMC5554187 28801572

[25] Bose SK, Mallinson JB, Gazoni RM, Brown SA (2017). Stable Self-Assembled Atomic-Switch Networks for Neuromorphic Applications. IEEE Trans. Electron Devices.

[26] Fostner S, Brown R, Carr J, Brown SA (2014). Continuum percolation with tunneling. Phys. Rev. B.

[27] Fostner S, Brown SA (2015). Neuromorphic behavior in percolating nanoparticle films. Phys. Rev. E - Stat. Nonlinear, Soft Matter Phys..

[28] Bose SK, Shirai S, Mallinson JB, Brown SA (2019). Synaptic dynamics in complex self-assembled nanoparticle networks. Faraday Discuss..

[29] Langley D (2013). Flexible transparent conductive materials based on silver nanowire networks: a review. Nanotechnology.

[30] Bellew AT, Manning HG, Gomes da Rocha C, Ferreira MS, Boland JJ (2015). Resistance of Single Ag Nanowire Junctions and Their Role in the Conductivity of Nanowire Networks. ACS Nano.

[31] Ye S, Rathmell AR, Chen Z, Stewart IE, Wiley BJ (2014). Metal Nanowire Networks: The Next Generation of Transparent Conductors. Adv. Mater..

[32] Sanguesa C, Urbina R, Figlarz M (1992). Synthesis and Characterization Particles of Uniform Shape of Fine and Monodisperse. J. Solid State Chem..

[33] Xia, Y. & Sun, Y. Shape-controlled synthesis of gold and silver nanoparticles. *Science.* **298**, 2176–2179 (2002). 10.1126/science.1077229 12481134

[34] Sun Y, Mayers B, Herricks T, Xia Y (2003). Polyol Synthesis of Uniform Silver Nanowires: A Plausible Growth Mechanism and the Supporting Evidence. Nano Lett..

[35] Murphy CJ, Jana NR (2002). Controlling the Aspect Ratio of Inorganic Nanorods and Nanowires. Adv. Mater..

[36] Bellew AT, Bell AP, McCarthy EK, Fairfield JA, Boland JJ (2014). Programmability of nanowire networks. Nanoscale.

[37] Sandouk, E. J., Gimzewski, J. K. & Stieg, A. Z. Multistate resistive switching in silver nanoparticle films. *Sci. Technol. Adv. Mater*. **16**, (2015). 10.1088/1468-6996/16/4/045004 PMC5090183 27877824

[38] Wu S (2011). A polymer-electrolyte-based atomic switch. Adv. Funct. Mater..

[39] White SI, Vora PM, Kikkawa JM, Winey KI (2011). Resistive Switching in Bulk Silver Nanowire-Polystyrene Composites. Adv. Funct. Mater..

[40] Nirmalraj PN (2012). Manipulating Connectivity and Electrical Conductivity in Metallic Nanowire Networks. Nano Lett..

[41] Manning, H. G. *et al*. Emergence of winner-takes-all connectivity paths in random nanowire networks. *Nat. Commun*. **9**, (2018). 10.1038/s41467-018-05517-6 PMC6089893 30104665

[42] Scharnhorst KS (2018). Atomic switch networks as complex adaptive systems. Jpn. J. Appl. Phys..

[43] Chialvo DR (2010). Emergent complex neural dynamics. Nat. Phys..

[44] Bak, P. (Per). *How nature works: the science of self-organized criticality*. (Copernicus, 1996).

[45] Fruchterman TMJ, Reingold EM (1991). Graph drawing by force-directed placement. Softw. Pract. Exp..

[46] Lu, W., Gaba, S., Sheridan, P., Zhou, J. & Choi, S.-H. Stochastic memristive devices for computing and neuromorphic applications. *Nanoscale* (2013). 10.1039/c3nr01176c 23698627

[47] Wang Z (2017). Memristors with diffusive dynamics as synaptic emulators for neuromorphic computing. Nat. Mater..

[48] Milotti, E. 1/f noise: a pedagogical review. *arXiv Prepr. physics/0204033*. (2002).

[49] Bak P, Tang C, Wiesenfeld K (1987). Self-organized criticality: An explanation of the 1/f noise. Phys. Rev. Lett..

[50] He BJ (2014). Scale-free brain activity: Past, present, and future. Trends Cogn. Sci..

[51] West BJ, Shlesinger M (1990). The Noise in Natural Phenomena. Am. Sci..

[52] Sannicolo T (2018). Electrical Mapping of Silver Nanowire Networks: A Versatile Tool for Imaging Network Homogeneity and Degradation Dynamics during Failure. ACS Nano.

[53] Bailey CH, Kandel ER, Harris KM (2015). Structural Components of Synaptic Plasticity and Memory Consolidation. Cold Spring Harb. Perspect. Biol..

[54] Reichold J (2009). Vascular graph model to simulate the cerebral blood flow in realistic vascular networks. J. Cereb. Blood Flow Metab..

[55] Corson F (2010). Fluctuations and redundancy in optimal transport networks. Phys. Rev. Lett..

[56] Katifori E, Szöllősi GJ, Magnasco MO (2010). Damage and Fluctuations Induce Loops in Optimal Transport Networks. Phys. Rev. Lett..

[57] Gräwer J, Modes CD, Magnasco MO, Katifori E (2015). Structural self-assembly and avalanchelike dynamics in locally adaptive networks. Phys. Rev. E.

[58] Martens, E. A. & Klemm, K. Transitions from Trees to Cycles in Adaptive Flow Networks. *Front. Phys*. **5**, (2017).

[59] Hesse, J. & Gross, T. Self-organized criticality as a fundamental property of neural systems. *Front. Syst. Neurosci*. **8**, (2014). 10.3389/fnsys.2014.00166 PMC4171833 25294989

[60] He BJ, Zempel JM, Snyder AZ, Raichle ME (2010). The temporal structures and functional significance of scale-free brain activity. Neuron.

[61] Marshall, N. *et al*. Analysis of Power Laws, Shape Collapses, and Neural Complexity: New Techniques and MATLAB Support via the NCC Toolbox. *Front. Physiol*. **7**, (2016). 10.3389/fphys.2016.00250 PMC4921690 27445842

[62] Beggs, J. M. & Timme, N. Being critical of criticality in the brain. *Front. Physiol*. 3 JUN (2012). 10.3389/fphys.2012.00163 PMC3369250 22701101

[63] Bi Y, Lu G (2008). Morphology-controlled Preparation of Silver Nanocrystals and Their Application in Catalysis. Chem. Lett..

[64] Nakayama T (2012). Development and application of multiple-probe scanning probe microscopes. Adv. Mater..

