# Supplementary material for: Emergent dynamics of neuromorphic nanowire networks
Source: Sci Rep. 2019 Oct 17;9:14920. doi: 10.1038/s41598-019-51330-6 (PMC6797708; doi:10.1038/s41598-019-51330-6)
Supplement: Supplementary file 1 — Supplementary Information [file 41598_2019_51330_MOESM1_ESM.pdf]

SUPPLEMENTARY INFORMATION:
Emergent dynamics of neuromorphic nanowire networks.

Adrian Diaz-Alvarez[1*], Rintaro Higuchi[1], Paula Sanz-Leon[2], Ido Marcus[2], Yoshitaka Shingaya[1], Adam Z. Stieg[1,3], James K. Gimzewski[1,3,4], Zdenka Kuncic[2], and Tomonobu Nakayama[1,2,5].**

1 International Center for Material Nanoarchitectonics(WPI-MANA),National Institute for Materials Science (NIMS), 1-1 Namiki, Tsukuba, Ibaraki, 305-0044 Japan
2 Sydney Nano Institute and School of Physics, University of Sydney, Sydney, NSW 2006, Australia
3 California NanoSystems Institute (CNSI), University of California Los Angeles, 570 Westwood Plaza, Los Angeles, California, 90095, USA
4 Department of Chemistry and Biochemistry, University of California Los Angeles, 607 Charles E. Young Drive East, Los Angeles, California 90095, USA
5 Graduate School of Pure and Applied Sciences, University of Tsukuba, 1-1 Namiki, Tsukuba, Ibaraki, 305-0055, Japan

***diazalvarez.adrian@nims.go.jp , **nakayama.tomonobu@nims.go.jp**

1. Computational modeling of network dynamics.

We modelled the dynamics of single nanowire junctions and the electrical properties of the network using Matlab R2017b (Mathworks).

Our model is based on previous work on single junction dynamics in Ag_2S atomic switches[1]. In this model, it is assumed that a single junction acts as a resistive atomic switch. The resistance of this switch is considered to arise from the formation of a single Ag atomic bridge or filament building up across the junction formed by overlapping nanowires. The growth of this conductive filament is regulated by the amount of current flowing through the junction and the resistance of the junction decreases linearly until reaching a saturation value when the silver filament length is equal to the junction gap. In previous work[1], the relation between resistance and junction filament length (or width) is obtained following the classical model of the memristor[2]. In this model, junction resistance decreases linearly from a disconnected (or open) state of high resistance when junction filament length is below a given formation threshold, to a connected (or closed) state of low resistance whenever this threshold is reached[3]. Based on our experimental observations, we typically use three orders of magnitude for the open/closed resistance ratio and a filament length of 3 nm.

Following *Sillin et. al.*[1], we also considered a decay term for the Ag filament evolution that accounts for the inherent instability of the junctions once the current flow is removed or reduced. The junction lifetime was set to 1 s. However, this model neglects the sudden conductance drops that arise whenever filaments are broken, as the resistance decreases linearly with filament length, as well as the susceptibility of large current carrying junctions to break apart. To account for this effect, we consider that the junction is broken (switches from closed to open) whenever the filament length surpasses a given dissolution threshold length, equal or smaller than the formation threshold length (3 nm).

To model the network dynamics, single nanowires are scattered on a two-dimensional grid with uniform random orientation and position. Overlapping points between nanowires are considered as junctions, and the network is transformed to a graph representation by considering nanowires as nodes and junctions as edges. This way, the physical network is translated to an abstract graph representation in which the adjacency, or laplacian matrix, is readily obtained to solve Kirchhoff's current equations at each time step, thus giving the voltage distribution on every node of the network, as well as the current going through edges. Simulation is further carried out by evolving the state of individual junctions with these currents, after which electrical resistance of the network and individual edges are computed with the evolved junction state. In the present simulation, a current source and sink are placed on individual nanowires at either end of the network, with a bias voltage applied to reproduce the effect of the physical double-probe experimental measurement scheme.

2. Typical activation time series in Ag-PVP nanowire networks.

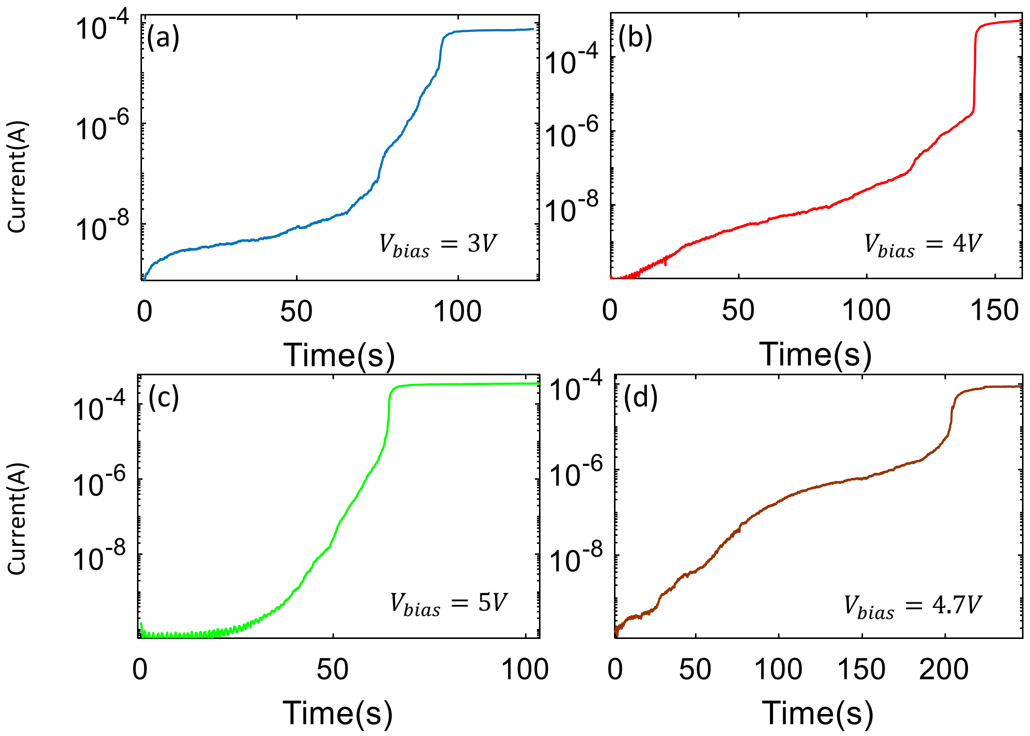

Figure S1: *a) to d): Archetypical activation time series measured from different Ag-PVP nanowire networks. Although all activation curves exhibit a similar sigmoidal shape, the time to activate the network as well as the threshold voltage are different. Interelectrode distance was the same in the four cases. Measurements taken with a Keithley-SMU. Current Compliance: 10mA.*

3. Analysis of current distribution in simulated network.

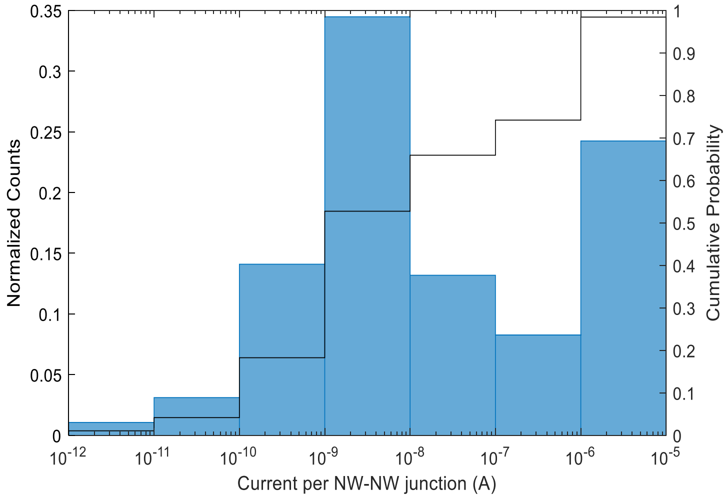

Figure S2: *Histograms (blue) showing the proportion of individual NW-NW junctions carrying a given current. Current axis is shown in log scale from pA to tenths of μA. The cumulative probability distribution is also shown (black line).*

4. Analysis of distribution of switching events in stochastic breakdown of junctions.

In Figs. S3a and S3b, we have obtained the distribution of time between consecutive plateaus from the complete set of measurements showing random decay (Fig. 3 of main text). As explained in the main text, the majority of decay traces drop in conductance immediately after switching the voltage from a super-threshold value to a sub-threshold value, with a percentage of curves decaying in irregular and step-like fashion with plateaus of different lengths and conductances (Fig. S3a). To further inspect the random nature of these events, we have considered that the onset of a drop in conductance followed by a plateau (switching event) is produced by a random junction breakdown event within the network. The distribution is shown in Fig S3b. Assuming a single energy barrier between junction formation and dissolution, we approximate the distribution to a Poisson distribution in which the probability of switching within time Δt at a time t is given by [4]:

$$P(t) = \Delta t/\tau \, e^{-\frac{t}{\tau}} \quad (1)$$

The fitted distribution follows the distribution of switching events with a typical time τ of 35.3s.

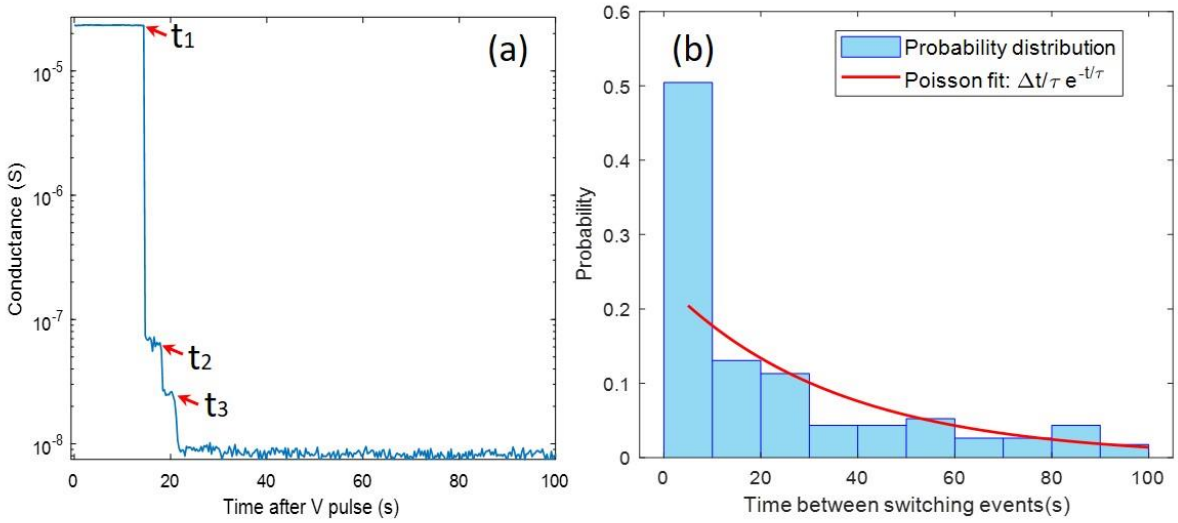

Figure S3: *a) Typical decay, or network deactivation profile acquired during 100 s after applying square pulses. The voltage applied to the network to probe decay state evolution was 10mV. In this example, red arrows point to the time that different decay switching events, which are followed by a conductance plateau, occurs. The distribution of the time between switching for a set of 100 decay curves has been plotted in figure b). The histogram bin size is equal to 10s. A red line, overlaid to the distribution, shows the fitted Poisson distribution of the data. A characteristic switching time, τ=35.3s, is observed.*

5. Comparison of beta distributions for other systems.

In Fig. S4, we compare the distribution of beta values when voltage magnitude and time of application are varied randomly for a PVP-Ag network, as well as for a fixed unique resistor with a value of 500 kΩ attached in series to the measurement and amplification system instead of the network. In the latter case (Figs. S4 (a) and (b)) the behavior of the resistor is perfectly ohmic, with very small fluctuations. The resulting beta magnitudes are approximately zero, consistent with thermal (white) noise from the resistor and measurement system. Fig. S4(c), shows the measured current time series for a PVP-Ag network under the influence of randomly varying voltage and acquisition times, in contrast to the measurement scheme described in the main body of the text, for which the voltage was linearly ramped up/down in small voltage steps and the time was kept fixed for every voltage value. In Fig. S4 (d), the beta magnitude distribution is similar to Fig. 5 (e), but slightly shifted towards larger values, as well as with a more pronounced tail at large beta values (close to 2). Thus, by randomly changing the voltage we remove the "feedback" effect persistent when voltage is slightly reduced (which does not compromise the transport backbone of the network) to produce more randomly fluctuating dynamics.

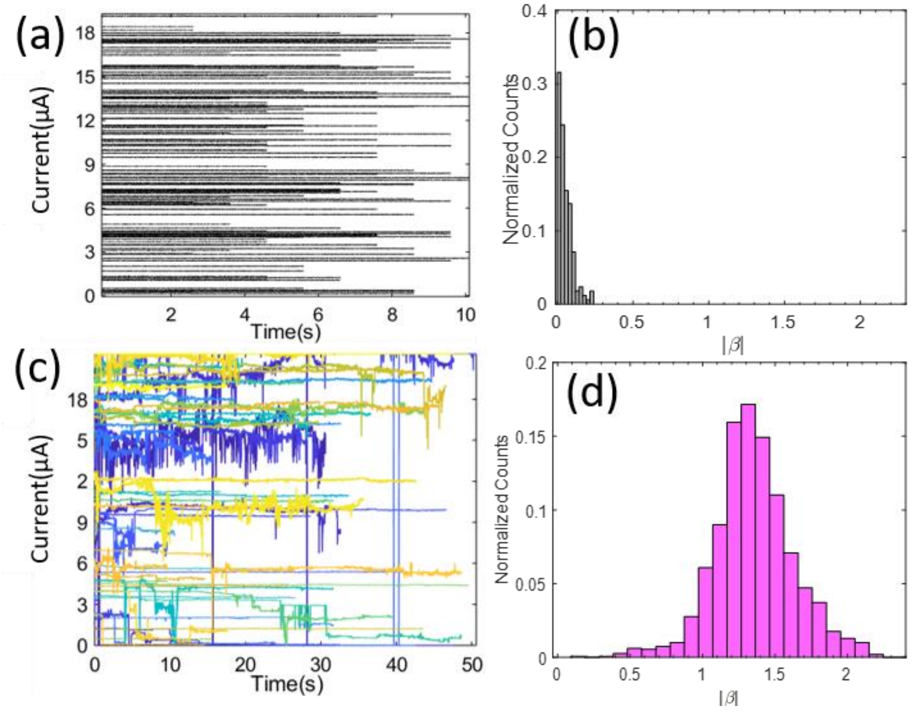

Figure S4: *a) Time series of current acquired in a 500 kΩ resistor with varying random voltages (from 0 to 3 V) and times (from 0 to 10 s). All the series are stacked in the same graph, and shifted to the origin for clarity. b) Distribution of beta magnitudes obtained from the slope of a power-law fit to the PSD computed from every time series (a). c) Current time series obtained from a PVP-Ag nanowire network with varying random voltages (from 0 to 3 V) and times (from 0 to 50 s). d) Beta magnitude distribution obtained from the PSDs in (c).*

References

1. Sillin, H. O. *et al.* A theoretical and experimental study of neuromorphic atomic switch networks for reservoir computing. *Nanotechnology* **24**, 384004 (2013).
2. Strukov, D. B., Snider, G. S., Stewart, D. R. & Williams, R. S. The missing memristor found. *Nature* **453**, 80–83 (2008).
3. Kuncic, Z. *et al.* Emergent brain-like complexity from nanowire atomic switch networks : Towards neuromorphic synthetic intelligence. *2018 IEEE 18th Int. Conf. Nanotechnol.* 1–3 (2018). doi:NANO.2018.8626236
4. Lu, W., Gaba, S., Sheridan, P., Zhou, J. & Choi, S.-H. Stochastic memristive devices for computing and neuromorphic applications. *Nanoscale* (2013).
